# One-Year Outcome of Intravitreal Tissue Plasminogen Activator, Ranibizumab, and Gas Injections for Submacular Hemorrhage in Polypoidal Choroidal Vasculopathy

**DOI:** 10.3390/jcm11082175

**Published:** 2022-04-13

**Authors:** Yorihisa Kitagawa, Hiroyuki Shimada, Ryusaburo Mori, Koji Tanaka, Yu Wakatsuki, Hajime Onoe, Hiroyuki Kaneko, Yumiko Machida, Hiroyuki Nakashizuka

**Affiliations:** Department of Ophthalmology, School of Medicine, Nihon University, 1-6 Surugadai, Kanda, Chiyodaku, Tokyo 101-8309, Japan; k.yorihisa@gmail.com (Y.K.); ryu-m@sa2.so-net.ne.jp (R.M.); taijinagaoka@gmail.com (K.T.); wakatsuki.yu@nihon-u.ac.jp (Y.W.); onoe.hajime@nihon-u.ac.jp (H.O.); kaneko.hiroyuki@nihon-u.ac.jp (H.K.); machida.yumiko@nihon-u.ac.jp (Y.M.); nkshizuk@gmail.com (H.N.)

**Keywords:** polypoidal choroidal vasculopathy, recombinant tissue plasminogen activator, ranibizumab, gas injections, aflibercept, central retinal thickness, univariate analyses, central pigment epithelial detachment thickness, multivariate analyses, recurrence, complications, submacular hemorrhage, pro re nata

## Abstract

This study investigated one-year outcomes of treatment with one session of intravitreal recombinant tissue plasminogen activator, ranibizumab, and gas injections for submacular hemorrhage secondary to polypoidal choroidal vasculopathy (PCV). An extended study of a previous prospective trial of this treatment modality in PCV patients was conducted in 64 patients (64 eyes). Early Treatment Diabetic Retinopathy Study (ETDRS) score, central retinal thickness (CRT), and central pigment epithelial detachment thickness (CPEDT) before and 1, 3, and 12 months after treatment were analyzed. Mean ETDRS score increased from 58 at baseline to 64 letters (*p* = 0.0122), CRT decreased from 543 to 192 μm (*p* < 0.0001), and CPEDT decreased from 161 to 103 μm (*p* = 0.0668) at 3 months and were maintained until 12 months. Complications requiring reoperation occurred within one month in four eyes. Recurrence was observed in 46 eyes (72%), and 1.6 ± 1.5 (0–7) intravitreal aflibercept injections were given pro re nata. Univariate and multivariate analyses identified CPEDT as the pre- and post-treatment factor affecting 12-month ETDRS score (*p* < 0.0001). Improved visual acuity stabilized 3 months after treatment. Although 72% of patients experienced recurrence, an average of 1.6 aflibercept injections/patient maintained visual acuity up to 12 months. CPEDT was the most important factor associated with visual outcome.

## 1. Introduction

Submacular hemorrhage is a serious complication of neovascular age-related macular degeneration (nAMD) or polypoidal choroidal vasculopathy (PCV) and leads to severe and irreversible damage to the photoreceptors and the outer nuclear layer [1,2]. PCV is a subtype of nAMD, commonly seen in Asian populations [3]. Among the presumed nAMD cases, the prevalence of PCV was 7.8% in the United States of America [4] and 23.0% in Japan [5]. In Asian patients, the incidence of submacular hemorrhage was higher in PCV (23.6%) than in nAMD (9.4%) [6], and submacular hemorrhage secondary to PCV may lead to chorioretinal atrophy and permanent vision loss [7,8,9]. The mean final visual acuity after follow-up of 23.9 ± 23.9 months was generally poor in patients with submacular hemorrhage secondary to nAMD (20/618) or PCV (20/240) [10]. For this reason, early treatment of submacular hemorrhage is necessary. Until recently, intravitreal recombinant tissue plasminogen activator (rt-PA) injection without vitrectomy (“nonvitrectomizing techniques”) [11,12,13,14,15] and submacular rt-PA injection with vitrectomy (“vitrectomizing techniques”) [16,17,18,19,20,21,22] are used as treatments for submacular hemorrhage. In both techniques, gas injection and/or anti-vascular endothelial growth factor (VEGF) agents can be added. Several studies have reported that both techniques have an acceptable safety profile and improve visual acuity and/or morphology [23,24,25,26,27]. There are no differences between the nonvitrectomizing and vitrectomizing techniques with respect to the postoperative response rate of complete submacular hemorrhage displacement and the complication rates of recurrent submacular hemorrhage and vitreous hemorrhage [28].

Currently, there is no consensus on the standard nonvitrectomizing treatment for submacular hemorrhage secondary to nAMD. Anti-VEGF monotherapy and combination therapy of anti-VEGF drug with gas or rt-PA are used. Anti-VEGF monotherapy is effective for small-sized hemorrhages and combination therapy for more than medium-sized lesions [11]. Among the combination therapies, rt-PA with gas treatment has been reported to have better visual outcome than anti-VEGF with gas, indicating the importance of displacing the submacular hemorrhage [12]. On the other hand, posttreatment visual outcome was found to be better after intravitreal injections of rt-PA, bevacizumab, and gas, compared with rt-PA and gas [13]. Therefore, treating the underlying lesion with an anti-VEGF agent and displacing the submacular hemorrhage with rt-PA and gas is a rational approach [14]. Ranibizumab (Lucentis; Novartis Pharma AG, Basel, Switzerland; Genentech Inc., South San Francisco, CA, USA) is not cleaved or functionally compromised by rt-PA or plasmin, whereas aflibercept (Eylea; Regeneron, Tarrytown, NY, USA, and Bayer HealthCare, Berlin, Germany) is cleaved and the VEGF-binding ability is reduced when co-administered with plasmin [29]. With this rationale, the authors previously conducted a prospective study to examine the efficacy of intravitreal injection of a combination of rt-PA, ranibizumab, and gas for treating subretinal hemorrhage in 19 PCV eyes followed for 6 months [14]. Although the authors demonstrated hemorrhage displacement and improved vision at 6 months posttreatment, the study had a small number of cases and a short follow-up. The authors therefore extended the study, recruiting more cases and prolonged the follow-up period to 12 months. This article reports our detailed analysis of the 1-year outcome focusing on visual acuity after treatment with one session of intravitreal rt-PA, ranibizumab, and gas injections for submacular hemorrhage in 64 PCV patients, as well as the pre- and post-treatment factors affecting visual outcome. Furthermore, nonvitrectomizing techniques and vitrectomizing techniques are discussed based on the results of this study and those reported in the literature.

## 2. Methods

### 2.1. Study Design and Subjects

The authors performed an extended study of a previous prospective study conducted from March 2014 to July 2015 at the Department of Ophthalmology of Nihon University Hospital [14]. In the extended study, the authors continued to recruit patients until September 2018 and analyzed all the patients recruited, including those analyzed in the previous prospective study [14] (study period: March 2014 to September 2018). The inclusion criteria were patients with thick submacular hemorrhage secondary to PCV, which involved the fovea with sizes of at least 2-disc diameters, and duration of symptoms was not more than 30 days. The exclusion criteria were underlying causes other than PCV, the presence of a macular scar, and cerebrovascular infarction or myocardial infarction within 3 months before the study receiving treatment. PCV was diagnosed by optical coherence tomography (OCT), fluorescein angiography, and indocyanine angiography as reported previously [7,8,9,10]. PCV was characterized by a complex choroidal vascular network with multiple terminal reddish-orange polypoidal lesions. When thick submacular hemorrhage hampered visualization of polypoidal lesion on indocyanine angiography, PCV was identified by characteristic OCT findings, including sharp-peaked pigment epithelial detachment [30].

### 2.2. Treatment and Examinations

All patients were treated with one session of intravitreal injections of rt-PA (Alteplase; Kyowa Hakko Kirin, Tokyo, Japan), ranibizumab, and perfluoropropane (C_3_F_8_; Alcon Laboratories Inc., Fort Worth, TX, USA) in an outpatient room, and then admitted for 3 days [14]. The patients were placed in a sitting position for 2 h, and then maintained in a prone position for 2 days.

The patients were examined before treatment daily from 1 to 3 days after treatment (as inpatient), at 2 weeks, and then monthly from 1 to 12 months (as outpatient). All patients underwent visual acuity measurement, intraocular pressure (IOP) measurement, slit-lamp biomicroscopy, indirect ophthalmoscopy, and OCT (Heidelberg Spectralis; Heidelberg Engineering Inc., Heidelberg, Germany) examination before and monthly from 1 to 12 months after treatment. Fluorescein angiography and indocyanine angiography were performed before and 1, 3, and 12 months after treatment.

Recurrence of submacular hemorrhage was evaluated by observation for rebleeding on color fundus photographs, and by measuring central retinal thickness (CRT) and central pigment epithelial detachment thickness (CPEDT) on OCT image as markers for early detection of recurrence [14]. Intravitreal injection of aflibercept was performed pro re nata (PRN) when submacular hemorrhage, serous retinal detachment, or mild vitreous hemorrhage was detected.

### 2.3. Outcome Measures

The primary outcome measure was Early Treatment Diabetic Retinopathy Study (ETDRS) score at 12 months after treatment. The secondary outcome measures were displacement of submacular hemorrhage; changes in CRT and CPEDT after treatment; complications; number of intravitreal injections administered during follow up and recurrence.

### 2.4. Intravitreal Tissue Plasminogen Activator, Ranibizumab and Gas Injections

All the intravitreal injections were conducted by one ophthalmologist (Y.K.). The procedures were performed in an outpatient injection room, which is separated from the outpatient clinic and equipped with a microscope and a bed. After administering retrobulbar anesthesia using 4 mL of 2% xylocaine, the eyelid skin and conjunctiva were disinfected with 0.25% povidone-iodine, prepared by diluting 10% povidone-iodine (Otsuka Pharmaceutical Co., Ltd., Tokyo, Japan) with physiological saline [31]. Then the eye was draped, and a lid speculum was placed. After performing paracentesis to remove 0.3 mL of aqueous humor, ranibizumab (0.5 mg/0.05 mL), rt-PA (25 μg/0.05 mL, 40,000 IU) and 100% perfluoropropane (C_3_F_8_; 0.3 mL) were injected intravitreally through the pars plana, successively in the same session. All three injections were performed using 30-gauge needles. The ocular surface was disinfected with several ml of 0.25% povidone-iodine before draping, and immediately before and after injection.

### 2.5. Measurements of Clinical Parameters

The greatest diameter of subretinal hemorrhage was measured. Displacement of submacular hemorrhage was evaluated on a fundus photograph taken 1 week after treatment. Complete displacement was defined as no blood or only a scant amount of blood within 1 disc diameter of the fovea. Partial displacement was defined as blood under the fovea that obscured the retinal pigment epithelium, but did not cause clinically visible elevation of the retina [14].

Recurrence of the hemorrhagic lesion was detected by the presence of rebleeding on color fundus photographs. As parameters for early detection of recurrence, CRT and CPEDT were also measured on OCT [14]. Surgical complications requiring re-operation occurring within one month after treatment were recorded. The number of intravitreal injections administered during the 12-month follow-up period (excluding the first injection for primary treatment) was counted.

### 2.6. Statistical Analyses

Statistical analyses were performed using SPSS software version 21 (SPSS, Inc., Chicago, IL, USA). Data are expressed as mean ± standard deviation (SD). A paired *t*-test was used to compare two groups. Univariate analysis (Spearman’s rank correlation coefficient [r]) and multivariate analysis were used to analyze the factors associated with visual acuity at 12 months after treatment. *p* values less than 0.05 were considered to be statistically significant.

## 3. Results

### 3.1. Baseline Data

A total of 64 eyes of 64 subjects (17 women and 47 men; mean age 72 ± 10 years, range, 48–91 years) were studied. The mean ± SD interval from onset of subretinal hemorrhage symptoms to treatment was 7 ± 7 (range, 1–30) days. The greatest diameter of submacular hemorrhage was 8 ± 6 (range, 2–27) disc diameters. The ETDRS score before treatment was 58 ± 19 (range, 5–84) letters (Table 1). The CRT was 543 ± 249 (range, 168–1276) μm, and CPEDT was 161 ± 227 (range, 0–837) μm.

### 3.2. Primary Outcome Measure

The ETDRS score was 64 ± 22 (range, 10–91) letters at 12 months after treatment and was significantly better than pretreatment visual acuity (*p* = 0.0105) (Table 1).

### 3.3. Secondary Outcome Measures

At 1 week after treatment, complete displacement of subretinal hemorrhage was observed in 49 eyes (77%) and partial displacement in 15 eyes (23%). At 12 months after treatment, CRT was 185 ± 103 (range, 76–720) μm, showing significant improvement compared to the pretreatment thicknesses (*p* < 0.0001). However, CPEDT was 110 ± 235 (range, 0–1366) μm, and was not significantly different from the pretreatment thickness (*p* = 0.1163). As supporting data, central pigment epithelial detachment (CPED) was present in 36 eyes (56%) and CPED was absent in 28 eyes (44%) before treatment. At 12 months after treatment, CPED was present in 26 eyes (41%) and was absent in 38 eyes (59%). There was no significant difference between the pre- and post-treatment periods (*p* = 0.4375).

Mean ETDRS score and mean CRT improved significantly at 3 months after treatment compared with before treatment (*p* = 0.0122 and *p* < 0.0001, respectively), but mean CPEDT did not improve significantly (*p* = 0.0668). However, there were no significant differences in mean ETDRS score, mean CRT, and mean CPEDT when comparing 3 and 12 months after treatment (*p* = 0.5659, 0.5003 and 0.5655, respectively). These results showed that treatment outcomes of improved visual acuity and CRT stabilized at 3 months after the procedure and were maintained up to 12 months after treatment.

Regarding posttreatment complications within one month after treatment, rhegmatogenous retinal detachment was found in 1 eye after 1 month, and vitreous hemorrhage was observed in 1 eye after 3 days and in 2 eyes after 13 days. Retinal detachment and vitreous hemorrhage were treated by vitrectomy using the Constellation system. Comparing pretreatment with 12-month posttreatment data in these 4 eyes, the ETDRS score and CRT improved significantly (*p* = 0.0459 and 0.0399, respectively), but CPEDT did not improve (*p* = 0.1879).

Recurrence was observed during 12 months of follow up in 46 eyes (72%); serous retinal detachment was detected in 25 eyes, subretinal hemorrhage in 12 eyes, vitreous hemorrhage in 7 eyes, and hemorrhagic PED in 2 eyes. Three of 46 eyes underwent pneumatic displacement of the submacular hemorrhage. These 46 patients received intravitreal injection of aflibercept PRN, and the mean number of injections was 1.6 ± 1.5 per patient. Comparing pretreatment with 12-month posttreatment data in these 46 eyes, the ETDRS score and CRT improved (*p* < 0.0001 and *p* < 0.0001, respectively), but CPEDT did not improve (*p* = 0.3973). In the supporting data, there was a correlation between the number of intravitreal injections at 12 months and pretreatment CPEDT (*p* = 0.0473).

**Figure 1:**
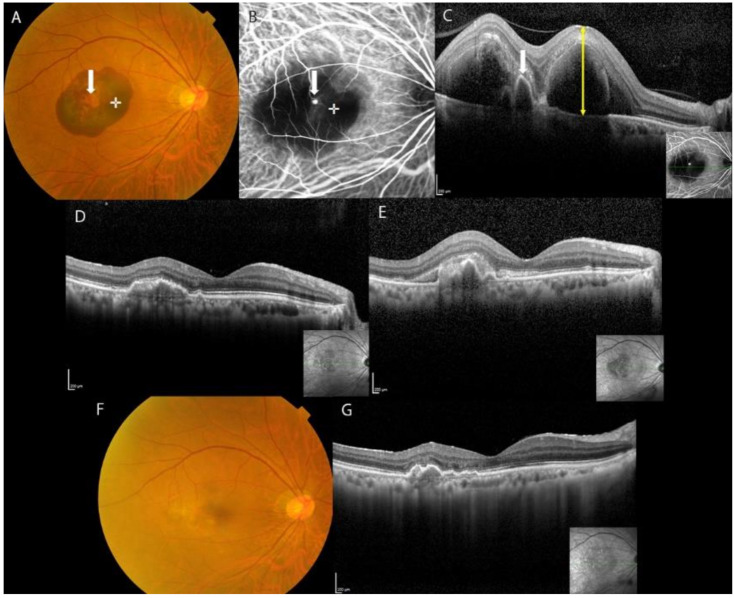
Image findings during the clinical course of a 65-year-old man with submacular hemorrhage secondary to polypoidal choroidal vasculopathy treated with intravitreal tissue plasminogen activator, ranibizumab and gas injections. Duration from onset to treatment was 4 days. (**A**) Before treatment, color fundus photograph shows submacular hemorrhage measuring 4 disc diameters involving the fovea. Reddish-orange polypoidal lesions (arrow) and the fovea (cross) are shown. (**B**) Pretreatment indocyanine green angiography shows a polypoidal lesion (arrow) and the fovea (cross). (**C**) Before treatment (ETDRS score 62 letters), optical coherence tomographic (OCT) image shows a polypoidal lesion with sharp-peaked pigment epithelial detachment (PED) (arrow). The central retinal thickness (CRT) is 642 μm (yellow up-down arrow), and there is no PED in the fovea (CPEDT was 0). (**D**) At 3 months after treatment (ETDRS score 73 letters), OCT image shows CRT of 165 μm and CPEDT of 0 μm. (**E**) At 4 months after treatment, OCT shows a subretinal hemorrhage recurring at the macula. An intravitreal injection of aflibercept was performed. (**F**) At 12 months after treatment, fundus photograph shows the disappearance of submacular hemorrhage. There was only one recurrence in 12 months. (**G**) At 12 months after treatment (ETDRS score 85 letters), OCT image shows CRT of 163 μm and CPEDT of 0 μm. In this case, good postoperative visual acuity was due to the absence of PED at the fovea before treatment. Central ellipsoid zone was well preserved. ETDRS: Early Treatment Diabetic Retinopathy Study, CPEDT: central pigment epithelial detachment thickness.

**Figure 2:**
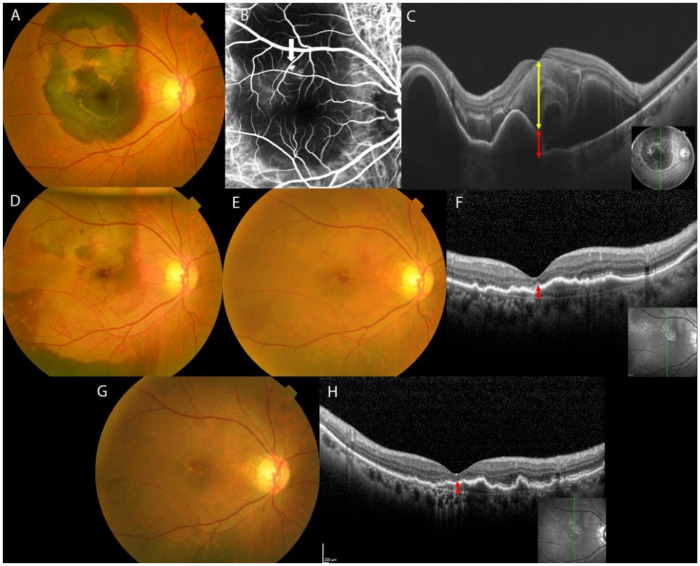
Image findings during the clinical course of a 64-year-old man with submacular hemorrhage secondary to polypoidal choroidal vasculopathy treated with intravitreal tissue plasminogen activator, ranibizumab and gas injections. Duration from onset to treatment was 2 days. (**A**) Before treatment, color fundus photograph shows submacular hemorrhage measuring 7 disc diameters involving the fovea. (**B**) Pretreatment indocyanine green angiography shows a polypoidal lesion (arrow). (**C**) Before treatment (ETDRS score 37 letters), optical coherence tomographic (OCT) shows central retinal thickness (CRT) of 795 μm (yellow up-down arrow) and CPEDT of 348 μm (red up-down arrow). (**D**) At 1 week after treatment, complete displacement of the submacular hemorrhage is confirmed. (**E**) At 3 months after treatment, the submacular hemorrhage has disappeared. (**F**) At 3 months after treatment (ETDRS score 77 letters), OCT image shows CRT of 116 μm and CPEDT of 70 μm. (**G**) At 12 months after treatment, fundus photograph shows no recurrence of submacular hemorrhage. (**H**) At 12 months after treatment (ETDRS score 51 letters), OCT image shows CRT of 111 μm and CPEDT of 79 μm. In this case, pigment epithelial detachment under the fovea was present before and after treatment, resulting in only mild ETDRS improvement. The central ellipsoid zone was not visible. ETDRS: Early Treatment Diabetic Retinopathy Study, CPEDT: central pigment epithelial detachment thickness.

Figure 1 and Figure 2 illustrate the clinical courses of two representative cases.

### 3.4. Injection-Related Adverse Events

In the present study, there were no ocular and no systemic adverse events associated with intravitreal rt-PA, ranibizumab and gas injections, such as systemic thromboembolic event and endophthalmitis.

### 3.5. Factors Affecting Pre-/Post-Treatment Visual Acuity

Univariate analysis of pretreatment factors showed that patients with thinner CPEDT (r = −0.499, *p* = 0.0142), better ETDRS score (r = +0.509, *p* = 0.0243), smaller subretinal hemorrhage (r = −0.226, *p* = 0.0315), and younger age (r = −0.379, *p* = 0.0417) had better ETDRS score at 12-month after treatment (Table 2). In multivariate analysis, the pretreatment factors found to be independently associated with ETDRS score at 12 months after treatment were pretreatment CPEDT (*p* < 0.0001), pretreatment ETDRS score (*p* < 0.0001), and age (*p* = 0.0020) (Table 2). Other variables comprising diameter of submacular hemorrhage (*p* = 0.0671), CRT (*p* = 0.1860), gender (*p* = 0.3640), and duration from onset to treatment (*p* = 0.4447) showed no association.

Univariate analysis of posttreatment factors showed that thinner 12-month CPEDT (r = −0.639, *p* = 0.0013), fewer posttreatment injections for recurrence (r = −0.370, *p* = 0.0045), complete displacement of subretinal hemorrhage (r = −0.422, *p* = 0.0084), no posttreatment complications (r = −0.379, *p* = 0.0179), and thinner posttreatment CRT (r = −0.242, *p* = 0.0324) had better ETDRS score at 12 months after treatment (Table 3). In multivariate analysis, the posttreatment factors found to be independently associated with ETDRS score at 12 months after treatment were 12-month CPEDT (*p* < 0.0001), displacement of submacular hemorrhage (*p* = 0.0005), number of posttreatment intravitreal injections (*p* = 0.0026), and posttreatment complications (*p* = 0.0404) (Table 3). However, CRT (*p* = 0.0527) showed no association.

From the correlation coefficients and the *p* values obtained from the above analyses, CPEDT was the most important pre- and post-treatment factor associated with posttreatment final visual acuity, and the thinner the CPEDT, the better was the ETDRS score at 12 months after treatment.

## 4. Discussion

In this study, when submacular hemorrhage secondary to PCV was treated with a single session of intravitreal rt-PA, ranibizumab and gas injections, vision improved and was stabilized 3 months after treatment, and although 72% of patients experienced recurrence, improved vision was maintained up to 12 months with an average of 1.6 intravitreal aflibercept injections per patient. This study also revealed that CPEDT was the most important pre- and post-treatment factor in predicting visual outcome. Ranibizumab was used concomitantly with rt-PA because this agent is not cleaved or functionally compromised by rt-PA or plasmin [29]. However, because polyp regression was more frequently observed with aflibercept treatment than with ranibizumab treatment [32], the authors used aflibercept for the management of posttreatment recurrence.

There is no published study that directly compares vitrectomizing and nonvitrectomizing techniques. Therefore, the authors examined nonvitrectomizing techniques and vitrectomizing techniques based on the present results and findings reported in the literature (Table 4). In an animal study that compared the pharmacokinetics of intravitreal anti-VEGF injections in aqueous humor of vitrectomized and non-vitrectomized macaque eyes [33], the half-lives of ranibizumab in aqueous humor of nonvitrectomized and vitrectomized eyes (2.3 and 1.4 days, respectively) were comparable to those of aflibercept (2.2 and 1.5 days). Both drugs had shorter half-lives in vitrectomized than in nonvitrectomized eyes. VEGF concentration decreased to undetectable level at 3 weeks in nonvitrectomized eyes and 1 week in vitrectomized eyes after ranibizumab injection, and at 6 weeks and 4 weeks after aflibercept injection. Therefore, the half-life of intravitreally injected ranibizumab or aflibercept was reduced to 60–70% in vitrectomized macaque eyes compared to nonvitrectomized eyes.

Extrapolating this data to patients, the difference in half-life of anti-VEGF agents in vitrectomized and nonvitrectomized eyes is expected to affect the number of posttreatment intravitreal anti-VEGF injections given PRN for the management of recurrent submacular hemorrhage in PCV or AMD. For submacular hemorrhage associated with PCV, the present study using a nonvitrectomizing technique required 2.6 ± 1.5 intravitreal anti-VEGF injections (including primary treatment) in 12 months. On the other hand, in a study using a vitrectomizing technique (vitrectomy with subretinal rt-PA and air tamponade), 4.1 ± 2.1 intravitreal anti-VEGF injections were administered PRN in 12 months [20]. For submacular hemorrhage secondary to nAMD, a study using non-vitrectomy techniques (gas + rtPA in 32 eyes, and bevacizumab + gas in 13 eyes) administered an average of 3.5 anti-VEGF injections in 12 months [12]. In comparison, a study using vitrectomizing technique (vitrectomy with subretinal co-application of rt-PA and bevacizumab and intravitreal gas tamponade) administered 4.5 (2 to 9) intravitreal anti-VEGF injections PRN during 12 months after surgery [18]. The larger number of posttreatment intravitreal anti-VEGF injections given in vitrectomizing techniques compared to nonvitrectomizing techniques may be related to the shorter half-lives of these agents in vitrectomized eyes.

Both nonvitrectomizing technique and vitrectomizing technique have been shown to be safe, and both improve visual acuity and morphology. The two techniques also have comparable postoperative outcome in terms of complete displacement rate, and similar postoperative complication rates including recurrence of submacular hemorrhage and vitreous hemorrhage [23,24,25,26]. The advantages of nonvitrectomizing technique are that there is no reduction in half-life of anti-VEGF agents in the vitreous because the vitreous is preserved, and that the procedure is simple and can be performed in an ophthalmology outpatient clinic, which is important in selecting a treatment method.

In our previous study with a smaller sample (19 eyes) followed for a shorter period (6 months), pre- and post-treatment central ellipsoid zone, pretreatment visual acuity, and pre- and post-treatment CPEDT were identified as important factors affecting posttreatment visual acuity [14]. However, the central ellipsoid zone was covered by subretinal hemorrhage in many eyes, and was evaluable in only 25% of the eyes in the present study. Therefore, the central ellipsoid zone was not analyzed as a variable in the present study. In the present extended study analyzing 64 eyes followed for 1 year, the authors conducted univariate and multivariate analyses to identify pretreatment factors associated with 12-month posttreatment ETDRS. Pretreatment CPEDT, ETDRS score, and age were identified as significant factors affecting 12-month postoperative ETDRS score. Pretreatment CPEDT was clearly the most important factor. Other variables such as diameter of submacular hemorrhage, gender, duration from onset to treatment, and CRT showed no association. This is probably because intravitreal injections of rt-PA, ranibizumab, and gas in combination effectively resolve subretinal hemorrhage and reduce CRT markedly, regardless of the size of subretinal hemorrhage or the time between onset and treatment.

In addition, univariate and multivariate analyses were performed to identify posttreatment factors impacting the ETDRS score at 12 months posttreatment. CPEDT at 12 months after treatment was also found to be the most important postoperative factor affecting the 12-month posttreatment ETDRS score. The reason that postoperative CPEDT remained a significant factor impacting posttreatment vision was that CPEDT of 1 μm or greater was found in 56% of the patients before treatment, and was still detected in 41% of the patients even after 12 months of treatment. These findings indicate that CPEDT associated with PCV is resistant to treatment and continues to affect vision at 12 months despite control of submacular hemorrhage by primary treatment and PRN anti-VEGF injection [34,35].

Posttreatment complications and recurrence affect postoperative vision and should be treated appropriately. Posttreatment complications requiring vitrectomy occurred within one month after treatment in four eyes, but with appropriate treatment, visual acuity at 12 months after treatment was significantly improved compared with before treatment. Recurrence was observed in 46 eyes (72%), and was managed with an average of 1.6 intravitreal aflibercept injections per patient. In these 46 eyes, ETDRS score and CRT at 12 months posttreatment improved significantly but CPEDT did not change. The number of intravitreal injections was higher in patients with thicker preoperative CPEDT, and CPED thickening tended to persist after treatment. Further randomized controlled clinical trials on nonvitrectomizing technique and vitrectomizing technique with a large number of cases and long-term follow-up are required.

This study has some limitations. First, all patients received intravitreal injections of ranibizumab, rt-PA and gas, based on the rationale that treating the underlying lesion with anti-VEGF agent and displacing the submacular hemorrhage with rt-PA and gas would optimize the treatment effect [11,12,13,14]. However, our study did not address which is the most effective agent or which combination therapy is the best option. Our treatment modality was designed for relatively large subretinal hemorrhages presented approximately one month after onset. On the other hand, in cases of small or fresh subretinal hemorrhages, monotherapy using anti-VEGF or gas injection may also be effective [11]. Further study is needed to examine appropriate treatment options for individual pathologic conditions. Second, in this study, the authors observed the course of treatment after the first intravitreal injection of anti-VEGF agent, with the main goal to compare with other treatment modalities. In the future, it is necessary to investigate whether three loading injections of anti-VEGF can further reduce recurrence.

In conclusion, when one session of intravitreal rt-PA, ranibizumab and gas injections was used as a non-vitrectomizing treatment for submacular hemorrhage secondary to PCV, visual acuity improved and stabilized three months after treatment. Although recurrence was observed in 72% of the patients during a follow up of one year, an average of 1.6 intravitreal aflibercept injections given PRN maintained the improved visual acuity until 12 months. Multivariate analysis identified CPEDT as the most important factor determining visual outcome.

## Figures and Tables

**Table 1:** Pre- and Post-Treatment ETDRS Scores and Central Retina Thickness.

	Pretreatment	Posttreatment			*p*-Value *			
	Pre	1M	3M	12M	Pre vs. 1M	Pre vs. 3M	Pre vs. 12M	3M vs. 12M
ETDRS score (letters)	58 ± 19	59 ± 21	64 ± 21	64 ± 22	0.6026	0.0122	0.0105	0.5659
CRT (μm)	543 ± 249	237 ± 130	192 ± 118	185 ± 103	<0.0001	<0.0001	<0.0001	0.5003
CPEDT (μm)	161 ± 227	111 ± 201	103 ± 237	110 ± 235	0.0549	0.0668	0.1163	0.5655

ETDRS: Early Treatment Diabetic Retinopathy Study, CRT: central retinal thickness, CPEDT: central pigment epithelial detachment thickness. * *p*-value by paired *t*-test.

**Table 2:** Analysis of Pretreatment Factors Associated with 12-Month Posttreatment ETDRS Score.

Parameter	Univariate Analysis		Multivariate Analysis *p*-Value
	r	*p*-Value	
Gender (female = 1, male = 2)	−0.115	0.2615	0.3640
Age (years)	−0.379	0.0417	0.0020
ETDRS score (letters)	+0.509	0.0243	<0.0001
CRT (μm)	−0.167	0.1227	0.1860
CPEDT (μm)	−0.499	0.0142	<0.0001
Duration from onset to treatment (days)	−0.097	0.3798	0.4447
Diameter of SMH (DD)	−0.226	0.0315	0.0671

ETDRS: Early Treatment Diabetic Retinopathy Study, CRT: central retinal thickness, CPEDT: central pigment epithelial detachment thickness, SMH: submacular hemorrhage, DD: disc diameters, r: Spearman’s rank correlation coefficient.

**Table 3:** Analysis of Posttreatment Factors Associated with 12-Month Posttreatment ETDRS Score.

Parameter	Univariate Analysis		Multivariate Analysis *p*-Value
	r	*p*-Value	
Displacement of SMH (complete = 1, partial = 2)	−0.422	0.0084	0.0005
Complications (No = 1, Yes = 2)	−0.379	0.0179	0.0404
Posttreatment intravitreal injection (number)	−0.370	0.0045	0.0026
12-Month CRT (μm)	−0.242	0.0324	0.0527
12-Month CPEDT (μm)	−0.639	0.0013	<0.0001

ETDRS: Early Treatment Diabetic Retinopathy Study, CRT: central retinal thickness, CPEDT: central pigment epithelial detachment thickness, SMH: submacular hemorrhage, r: Spearman’s rank correlation coefficient.

**Table 4:** Nonvitrectomizing Techniques and Vitrectomizing Techniques.

	Nonvitrectomizing Techniques	Vitrectomizing Techniques
Vitreous Status	Nonvitrectomized Eye	Vitrectomized Eye
Half-life of intravitreally injected ranibizumab and aflibercept (macaque eyes) [33]	Ranibizumab: 2.3 ± 0.2 days Aflibercept: 2.2 ± 0.1 days	Ranibizumab: 1.4 ± 0.6 days (reduced 60.9%) Aflibercept: 1.5 ± 0.3 days (reduced 68.2%)
Number of PRN intravitreal anti-VEGF injections for posttreatment recurrence of SMH in PCV *	2.6 ± 1.5 injections/patient during 12 months in 64 eyes (this study)	4.1 ± 2.1 injections/patient during 12 months in 23 eyes [20] ^†^
Number of PRN intravitreal anti-VEGF injections for posttreatment recurrence of SMH in nAMD *	3.5 injections/patient during 12 months in 45 eyes [12] **	4.5 injections/patient during 12 months in 41 eyes [18] ^††^
Safety profile, visual acuity improvement, complete displacement rate, and rates of recurrent SMH in nAMD	No differences between the two techniques [23,24,25,26]	

VEGF: vascular endothelial growth factor, PCV: polypoidal choroidal vasculopathy, PRN: pro re nata, SMH: submacular hemorrhage, nAMD: neovascular age-related macular degeneration, rt-PA: recombinant tissue plasminogen activator. * Number of intravitreal anti-VEGF injections in 12 months, including the primary treatment. ** Thirty-two eyes were treated with gas + rt-PA and 13 eyes with bevacizumab + gas. ^†^ Primary treatment: vitrectomy, submacular rt-PA, intravitreal air tamponade. ^††^ Primary treatment: vitrectomy, submacular rt-PA and bevacizumab, intravitreal gas tamponade.

## Data Availability

The datasets of this study are available from the corresponding author upon reasonable request.

## References

[1] Glatt H., Machemer R. (1982). Experimental subretinal hemorrhage in rabbits. Am. J. Ophthalmol..

[2] Toth C.A., Morse L.S., Hjelmeland L.M., Landers M.B. (1991). Fibrin directs early retinal damage after experimental subretinal hemorrhage. Arch. Ophthalmol..

[3] Wong C.W., Yanagi Y., Lee W.K., Ogura Y., Yeo I., Wong T.Y., Cheung C.M.G. (2016). Age-related macular degeneration and polypoidal choroidal vasculopathy in Asians. Prog. Retin. Eye Res..

[4] Yannuzzi L.A., Wong D.W., Sforzolini B.S., Goldbaum M., Tang K.C., Spaide R.F., Freund K.B., Slakter J.S., Guyer D.R., Sorenson J.A. (1999). Polypoidal choroidal vasculopathy and neovascularized age-related macular degeneration. Arch. Ophthalmol..

[5] Sho K., Takahashi K., Yamada H., Wada M., Nagai Y., Otsuji T., Nishikawa M., Mitsuma Y., Yamazaki Y., Matsumura M. (2003). Polypoidal choroidal vasculopathy: Incidence, demographic features, and clinical characteristics. Arch. Ophthalmol..

[6] Kim J.H., Chang Y.S., Kim J.W., Kim C.G. (2017). Characteristics of submacular hemorrhages in age-related macular degeneration. Optom. Vis. Sci..

[7] Yannuzzi L.A., Ciardella A., Spaide R.F., Rabb M., Freund K.B., Orlock D.A. (1997). The expanding clinical spectrum of idiopathic polypoidal choroidal vasculopathy. Arch. Ophthalmol..

[8] Uyama M., Matsubara T., Fukushima I., Matsunaga H., Iwashita K., Nagai Y., Takahashi K. (1999). Idiopathic polypoidal choroidal vasculopathy in Japanese patients. Arch. Ophthalmol..

[9] Uyama M., Wada M., Nagai Y., Matsubara T., Matsunaga H., Fukushima I., Takahashi K., Matsumura M. (2002). Polypoidal choroidal vasculopathy: Natural history. Am. J. Ophthalmol..

[10] Kim H., Lee S.C., Kim S.M., Lee J.H., Koh H.J., Kim S.S., Byeon S.H., Kim M., Lee C.S. (2015). Identification of underlying causes of spontaneous submacular hemorrhage by indocyanine green angiography. Ophthalmologica.

[11] Jeong S., Park D.G., Sagong M. (2020). Management of a submacular hemorrhage secondary to age-related macular degeneration: A Comparison of three treatment modalities. J. Clin. Med..

[12] Mayer W.J., Hakim I., Haritoglou C., Gandorfer A., Ulbig M., Kampik A., Wolf A. (2013). Efficacy and safety of recombinant tissue plasminogen activator and gas versus bevacizumab and gas for subretinal haemorrhage. Acta Ophthalmol..

[13] Guthoff R., Guthoff T., Meigen T., Goebel W. (2011). Intravitreous injection of bevacizumab, tissue plasminogen activator, and gas in the treatment of submacular hemorrhage in age-related macular degeneration. Retina.

[14] Kitagawa Y., Shimada H., Mori R., Tanaka K., Yuzawa M. (2016). Intravitreal tissue plasminogen activator, ranibizumab, gas Injection for submacular hemorrhage in polypoidal choroidal vasculopathy. Ophthalmology.

[15] Lee S.H., Lee S.J., Shin Y.I., Lim H.B., Kim J.Y., Han Y.S., Nam K.Y. (2021). The effect of initial intravitreal tissue plasminogen activator and gas injection on vision improvement in patients with submacular haemorrhage associated with age-related macular degeneration. Eye.

[16] Haupert C.L., McCuen B.W., Jaffe G.J., Steuer E.R., Cox T.A., Toth C.A., Fekrat S., Postel E.A. (2001). Pars plana vitrectomy, subretinal injection of tissue plasminogen activator, and fluid-gas exchange for displacement of thick submacular hemorrhage in age-related macular degeneration. Am. J. Ophthalmol..

[17] Olivier S., Chow D.R., Packo K.H., MacCumber M.W., Awh C.C. (2004). Subretinal recombinant tissue plasminogen activator injection and pneumatic displacement of thick submacular hemorrhage in age-related macular degeneration. Ophthalmology.

[18] Treumer F., Roider J., Hillenkamp J. (2012). Longterm outcome of subretinal coapplication of rtPA and bevacizumab followed by repeated intravitreal anti-VEGF injections for neovascular AMD with submacular haemorrhage. Br. J. Ophthalmol..

[19] Chang W., Garg S.J., Maturi R., Hsu J., Sivalingam A., Gupta S.A., Regillo C.D., Ho A.C. (2014). Management of thick submacular hemorrhage with subretinal tissue plasminogen activator and pneumatic displacement for age-related macular degeneration. Am. J. Ophthalmol..

[20] Kimura S., Morizane Y., Hosokawa M., Shiode Y., Kawata T., Doi S., Matoba R., Hosogi M., Fujiwara A., Inoue Y. (2015). Outcomes of vitrectomy combined with subretinal tissue plasminogen activator injection for submacular hemorrhage associated with polypoidal choroidal vasculopathy. Am. J. Ophthalmol..

[21] Kadonosono K., Arakawa A., Yamane S., Inoue M., Yamakawa T., Uchio E., Yanagi Y. (2015). Displacement of submacular hemorrhages in age-related macular degeneration with subretinal tissue plasminogen activator and air. Ophthalmology.

[22] Treumer F., Wienand S., Purtskhvanidze K., Roider J., Hillenkamp J. (2017). The role of pigment epithelial detachment in AMD with submacular hemorrhage treated with vitrectomy and subretinal co-application of rtPA and anti-VEGF. Graefes Arch. Clin. Exp. Ophthalmol..

[23] Stanescu-Segall D., Balta F., Jackson T.L. (2016). Submacular hemorrhage in neovascular age-related macular degeneration: A synthesis of the literature. Surv. Ophthalmol..

[24] Fassbender J.M., Sherman M.P., Barr C.C., Schaal S. (2016). Tissue plasminogen activator for subfoveal hemorrhage due to age-related macular degeneration: Comparison of 3 treatment modalities. Retina.

[25] Grohmann C., Dimopoulos S., Bartz-Schmidt K.U., Schindler P., Katz T., Spitzer M.S., Skevas C. (2020). Surgical management of submacular hemorrhage due to n-AMD: A comparison of three surgical methods. Int. J. Retin. Vitr..

[26] De Jong J.H., van Zeeburg E.J., Cereda M.G., van Velthoven M.E., Faridpooya K., Vermeer K.A., van Meurs J.C. (2016). Intravitreal versus subretinal administration of recombinant tissue Plasminogen activator combined with gas for acute submacular hemorrhages due to age-related macular degeneration. An exploratory prospective study. Retina.

[27] Tranos P., Tsiropoulos G.N., Koronis S., Vakalis A., Asteriadis S., Stavrakas P. (2021). Comparison of subretinal versus intravitreal injection of recombinant tissue plasminogen activator with gas for submacular hemorrhage secondary to wet age-related macular degeneration: Treatment outcomes and brief literature review. Int. Ophthalmol..

[28] Van Zeeburg E.J., van Meurs J.C. (2013). Literature review of recombinant tissue plasminogen activator used for recent-onset submacular hemorrhage displacement in age-related macular degeneration. Ophthalmologica.

[29] Klettner A., Grotelüschen S., Treumer F., Roider J., Hillenkamp J. (2015). Compatibility of recombinant tissue plasminogen activator (rtPA) and aflibercept or ranibizumab coapplied for neovascular age-related macular degeneration with submacular haemorrhage. Br. J. Ophthalmol..

[30] Cheung C.M.G., Lai T.Y.Y., Teo K., Ruamviboonsuk P., Chen S.J., Kim J.E., Gomi F., Koh A.H., Kokame G., Jordan-Yu J.M. (2021). Polypoidal choroidal vasculopathy: Consensus nomenclature and non-indocyanine green angiograph diagnostic criteria from the Asia-Pacific Ocular Imaging Society PCV Workgroup. Ophthalmology.

[31] Shimada H., Hattori T., Mori R., Nakashizuka H., Fujita K., Yuzawa M. (2013). Minimizing the endophthalmitis rate following intravitreal injections using 0.25% povidone-iodine irrigation and surgical mask. Graefes Arch. Clin. Exp. Ophthalmol..

[32] Cho H.J., Kim K.M., Kim H.S., Han J.I., Kim C.G., Lee T.G., Kim J.W. (2016). Intravitreal Aflibercept and Ranibizumab Injections for Polypoidal Choroidal Vasculopathy. Am. J. Ophthalmol..

[33] Niwa Y., Kakinoki M., Sawada T., Wang X., Ohji M. (2015). Ranibizumab and aflibercept: Intraocular pharmacokinetics and their effects on aqueous VEGF level in vitrectomized and nonvitrectomized macaque eyes. Investig. Ophthalmol. Vis. Sci..

[34] Inoue M., Arakawa A., Yamane S., Kadonosono K. (2013). Variable response of vascularized pigment epithelial detachments to ranibizumab based on lesion subtypes, including polypoidal choroidal vasculopathy. Retina.

[35] Baba T., Kitahashi M., Kubota-Taniai M., Oshitari T., Yamamoto S. (2012). Two-year course of subfoveal pigment epithelial detachment in eyes with age-related macular degeneration and visual acuity better than 20/40. Ophthalmologica.

